# Supplementary material for: Reconstruction of the X and Y haplotypes in the genetically improved Abbassa nile tilapia genome assembly
Source: Sci Rep. 2025 May 8;15:16057. doi: 10.1038/s41598-025-01300-y (PMC12062369; doi:10.1038/s41598-025-01300-y)
Supplement: Supplementary file 6 — Supplementary Material 6 [file 41598_2025_1300_MOESM6_ESM.docx]

**Supplementary Table 1.** Number of BUSCOs identified as complete, duplicated, fragmented or missing in the Abbassa annotation

| **Busco Plots (actinopterygii_odb10)** | **Abbassa annotation** |
| --- | --- |
| Complete (single copy) | 3566 |
| Complete (2 copies) | 56 |
| Complete (3 copies) | 4 |
| Complete (4+ copies) | 1 |
| Complete | 3627 |
| Duplicated | 61 |
| Fragmented | 3 |
| Missing | 10 |
| Total | 3640 |
